# Joint Awareness after Unicompartmental Knee Arthroplasty Evaluated with the Forgotten Joint Score

**DOI:** 10.1111/os.12613

**Published:** 2020-02-19

**Authors:** Yi‐ke Dai, Wei Lin, Guang‐min Yang, Jiang‐feng Lu, Fei Wang

**Affiliations:** ^1^ Department of Joint Surgery, Third Hospital of Hebei Medical University Shijiazhuang China

**Keywords:** Forgotten Joint Score (FJS), Unicompartmental knee arthroplasty (UKA), BMI, Age, Gender

## Abstract

**Objective:**

To investigate the temporal relationship of medial unicompartmental knee arthroplasty (UKA) and forgotten joint score (FJS), and to analysis the predictive factors associated with FJS after medial UKA.

**Methods:**

This is a cross‐sectional observational study. A total of 188 cases of medial UKA were included in this study, and all the prostheses used were Oxford mobile‐bearing UKA from January 2016 to January 2019. All patients have completed the questionnaire of FJS, and the relevant data were obtained for 1 month (n = 38), 6 month (n = 40), 12 month (n = 42), 24 month (n = 34), and 36 month (n = 34) patient subgroups. The score ranged from 0–100, with a higher score indicating a more natural knee joint. In addition, the associations between the potential influencing factors (body mass index [BMI], age, gender, duration of onset before surgery, Kellgren‐Lawrence grade of the medial compartment before surgery) with FJS were analyzed using Pearson correlation and multiple linear regression.

**Results:**

Postoperative FJSs were 44.5 ± 13.5 at 1 month，63.8 ± 10.1 at 6 months, 77.1 ± 12.2 at 12 months, 78.4 ± 12.2 at 24 months, 78.9 ± 12.5 at 36 months. The postoperative FJSs were lowest at 1 month and highest at 36 month (*P* < 0.01). The mean value of FJS kept improving until 12 months post‐operation, which was slightly lower than that of 24 months and 36 months, but there was no statistical difference between them. Pearson correlation and multiple linear regression analysis showed that gender and Kellgren‐Lawrence grade of the medial compartment before surgery had no significant influence on FJS, while age, BMI, and duration of onset before surgery had significant associations with FJS after UKA. BMI was negatively correlated with FJS, while older patients (>60) and with longer duration of onset before surgery (>3 years) were a positive predictor of good outcome for the FJS.

**Conclusion:**

Patients can expect marked improvement in the natural feel of the prosthesis during the first year after UKA, slight continued improvement at 2 and 3 years. Furthermore, we identified three preoperative patient‐related factors (age, BMI, and duration of onset before surgery) that may predict the FJS after medial UKA, which can be used to guide surgical decision making.

## Introduction

In the last 20 years, the utilization of unicompartmental knee arthroplasty (UKA) increased dramatically as an effective surgical method for the treatment of unicompartmental osteoarthritis. From 1998 to 2005, the utilization of UKA increased almost three times faster than total knee replacement due to less blood loss, higher range of motion after operation, preservation of bone mass and ligaments, and faster recovery^1^. A large number of researchers have shown that the survivorship rate of modern UKA prosthesis is greater than 90% and the rate of postoperative complications is relatively low over 10‐years follow‐up^2^, ^3^, ^4^. Currently, the assessment methods for the postoperative outcomes of joint replacement often focus on objective clinical evaluations such as postoperative activity, survival of prosthesis, complication and revision rate, which often ignore the subjective feelings of patients themselves, and may cause differences in postoperative focus between patients and surgeons^5^. Furthermore, these assessment methods show weakness in discriminating satisfactory, great, and exceptional outcomes. In our opinion, the ability to forget the artificial joint in daily life can be regarded as the ultimate goal in joint arthroplasty resulting in the greatest possible patient satisfaction.

The Forgotten Joint Score (FJS) is a scoring system developed in recent years and based on questionnaires of 12 different questions to understand the patients' ability to forget their artificial knee joint in daily life. From 0 to 100, the higher the score, the more natural or “forgotten” the joint. Furthermore, compared with other patient‐reported outcome measurements, FJS is not limited by the ceiling effect^6^. While the FJS has been extensively used in patients with total hip arthroplasty (THA) and total knee arthroplasty (TKA), less is known about patients undergoing medial UKA^7^, ^8^. Zuiderbaan *et al*. compared UKA with TKA and found that the FJS was significantly higher in the UKA group of patients in contrast to the TKA group; patients who undergo UKA are more likely to forget their artificial joint in daily life and consequently may be more satisfied^9^. This is similar to the results of research by Kim *et al*., who found that patients who underwent UKA had higher FJS, high flexion knee score (HFKS), and satisfaction rate when compared with patients who underwent TKA, indicating that UKA facilitated less knee awareness and better function and satisfaction than TKA^10^. However, these studies are all comparative studies between TKA and UKA, and the study related to the natural history of FJS for 3 years after medial UKA were not found.

In addition, it is well known that final outcome scores after joint replacement are correlated with pre‐intervention data. Therefore, identifying predictors of outcome after medial UKA is important, because this information can help patients have accurate expectations before surgery and also allows surgeons to risk‐stratify patients for outcomes, while searching for modifiable factors or interventions that might improve outcomes. For the FJS, there are rarely reports concerning this issue and it is unknown if predictors for the forgotten joint after medial UKA exist.

Therefore, the purpose of this study was to: (i) evaluate the patient reported outcomes (PROs) regarding joint awareness following UKA at defined intervals; and (ii) identify the associations between the potential influencing factors (BMI, age, gender, duration of onset before surgery, Kellgren‐Lawrence grade of the medial compartment before surgery) with FJS following medial UKA.

## Material and Methods

### *Patients*

This is a cross‐sectional observational study. A total of 188 cases of medial UKA were included in this study and all the prostheses used were Oxford mobile‐bearing UKA from January 2016 to January 2019. No patients were lost to follow‐up. The inclusion criteria were: (i) patients with complete medical records; (ii) patients undergoing primary unilateral medial unicompartmental knee arthroplasty; (iii) all patients signed the informed consent before surgery; and (iv) all surgeries were performed by the same surgeon in our institution. The patient records were reviewed, and the following exclusion criteria were applied: (i) revision arthroplasty and post‐infection; (ii) simultaneous or staged bilateral operation; (iii) the ipsilateral knee has a history of surgery; (iv) long‐term use of painkillers; (v) patients who could not cooperate with the completion of FJS score; and (vi) patients that underwent arthroscopy or other surgical procedures before surgery. Participants were only allowed to complete the survey once, i.e. no data was collected from a single patient for multiple follow‐up intervals in regard to the same knee.

### *Surgery*

All medial UKA surgeries were performed by the same surgeon in a hospital with over 10 years of experience in joint surgery. All UKA operations included were performed in strict accordance with the Oxford operating manual, and the surgical techniques remained consistent. A medial parapatellar approach was applied in all cases, about 10 cm in length.

### *Forgotten Joint Score*

FJS‐12 consists of 12 questions and is scored using a 5‐point Likert response format with the raw scores transformed onto a 0–100 point scale. Higher scores indicate a more favorable outcome, i.e. a more natural artificial joint. The FJS‐12 has been shown to have a low ceiling effect and discriminates well between good, very good, and excellent outcome after joint arthroplasty. All patients in our study were contacted by phone to complete the FJS‐12 questionnaire, and relevant data were obtained for 1 month, 6 month, 12 month, 24 month, and 36 month patient subgroups. In addition, to determine whether gender, body mass index (BMI) (>30 or <30), age (>60 or <60), duration of onset before surgery (>3 years or <3 years), and/or Kellgren and Lawrence grade (III or IV) of the medial compartment before surgery had an impact on the outcomes, we analyzed the associations between these potential influencing factors with FJS. This study was approved by the institutional review committee of our institute.

### *Statistical Analysis*

All data are represented by mean and standard deviation in our study. We evaluated homogeneity of variance by using Levene's test and Kolmogorov–Smirnov test was performed to assess normality. Repeated one‐way analysis of variance (ANOVA) was used to compare the FJS averages and the continuous demographic that confirmed homogeneity of variance and normal distribution. When the ANOVA test showed statistical significance, the Tukey *post hoc* test was used to identify the specific patient subgroups. Categorical demographic was compared by performing chi‐squared test. Possible associated influence factors to FJS included: gender, age, BMI, duration of onset before surgery, Kellgren‐Lawrence grade of the medial compartment before surgery. Pearson correlation and multiple linear regression were performed to analyze the correlation between FJS score (dependent variable) at each time point after surgery and all the predictor variables (independent variable). All statistical analyses were performed by using SPSS 21.0 (SPSS Inc., Chicago, IL, US). A *P* value <0.05 was considered as statistically significant.

## Results

### *Forgotten Joint Score (FJS) Outcomes*

**Figure 1:**
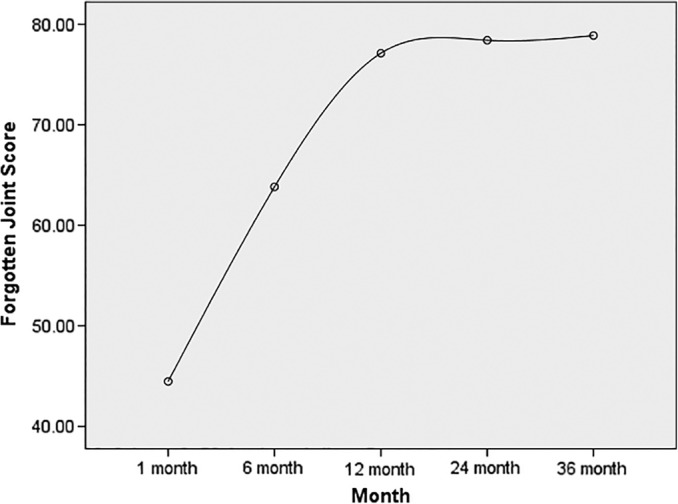
Mean value of Forgotten Joint Score at 1 month, 6 months, 1 year, 2 years and 3 years after UKA. Thirty‐six months after medial UKA demonstrated the highest forgotten joint score (FJS). A platform stage was shown from 12 month to 36 month following medial UKA.

All the 188 patients after UKA surgery completed the questionnaire, and the average BMI, age, and gender of patient cohorts is shown in Table 1. No patients underwent revision or reoperations during follow‐up. Postoperative FJSs were 44.5 ± 13.5 at 1 month，63.8 ± 10.1 at 6 months, 77.1 ± 12.2 at 12 months, 78.4 ± 12.2 at 24 months, and 78.9 ± 12.5 at 36 months. The postoperative FJSs were lowest at 1 month (44.5 ± 13.5, *P* < 0.01) and highest at 36 months (78.9 ± 12.5, *P* < 0.01). The FJS at 6 months after surgery (63.8 ± 10.1) dramatically improved, i.e. the rate of change was the greatest at 6 months after operation, which was significantly lower than that at 1 year after surgery (*P* < 0.01). The FJS kept improving till 12 months post‐operation (77.1 ± 12.2), which was slightly lower than that of 24 months (78.4 ± 12.2) and 36 months (78.9 ± 12.5), but there was no statistical difference between them (Fig. 1). So, that is to say, after 12 months improvement stopped.

**Table 1:** Patient Demographics

	1 month	6 month	12 month	24 month	36 month	*P* values
Number	38	40	42	34	34	n.s.
Age	66.8(10.6)	67.2(11.6)	66.6(10.5)	65.8(10.2)	67.2(11.2)	n.s.
Sex, males	20(52.6%)	18(45%)	20(47.6%)	19(55.8%)	14(42.2%)	n.s.
BMI	29.1(2.9)	28.6(4.2)	29.5(3.1)	28.4(4.0)	28.8(3.8)	n.s.
Duration (<3Y)	17(44.7%)	20(50%)	22(52.4%)	15(44.1%)	18(52.9%)	n.s.
K‐L grade(IV)	18(47.4%)	21(52.5%)	22(52.4%)	17(50%)	17(50%)	n.s.

n.s, no significant; BMI, body mass index; Duration, duration of onset before surgery; K‐L grade, Kellgren‐Lawrence grade; 3Y, 3 years

### *Predictive Factors Outcomes*

The Pearson correlation analysis results were shown in Table 2. The gender and Kellgren‐Lawrence grade of the medial compartment before surgery have no correlation with FJS score at each time point after UKA (*P* > 0.05). The age was positively correlated with FJS score from 12 months after UKA, while BMI and duration of onset was negatively correlated with FJS score from 12 months after UKA (*P* < 0.05).

**Table 2:** Pearson correlation analysis results of FJS and other factors at various time points after medial unicompartmental knee arthroplasty

	1 month		6 month		12 month		24 month		36 month	
	Correlation	*P*	Correlation	*P*	Correlation	*P*	Correlation	*P*	Correlation	*P*
Age	0.28	0.09	−0.224	0.18	0.769	0.00	0.695	0.00	0.755	0.00
Gender	0.10	0.96	0.016	0.93	0.114	0.49	−0.185	0.267	−0.067	0.689
BMI	0.024	0.89	−0.044	0.79	−0.500	0.001	−0.594	0.000	−0.637	0.000
Duration	0.189	0.26	0.204	0.22	0.645	0.000	0.644	0.002	0.676	0.002
K‐L grade	−0.018	0.92	−0.042	0.80	−0.093	0.58	0.041	0.81	−0.209	0.21

BMI, body mass index; K‐L grade, Kellgren‐Lawrence grade; FJS, Forgotten Joint Score; Duration, duration of onset before surgery.

**Figure 2:**
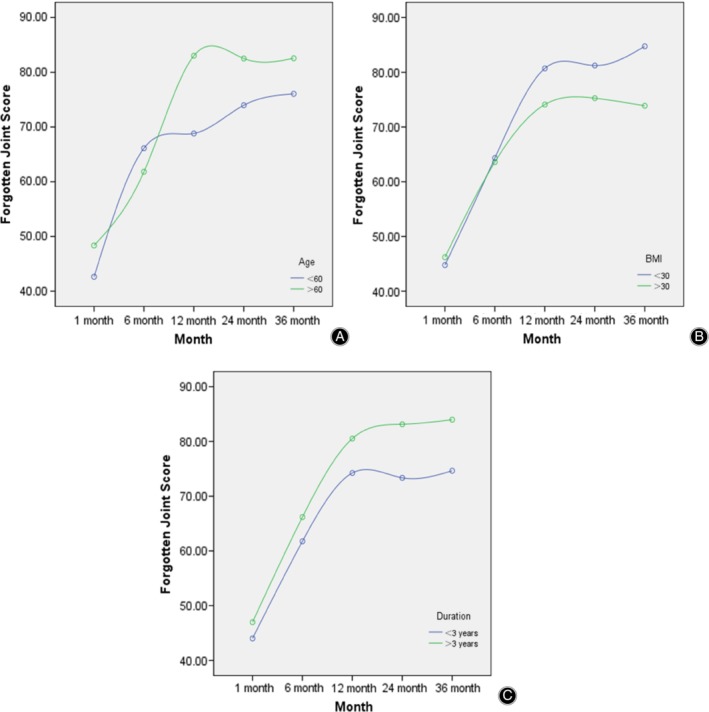
(A) Changes of Forgotten Joint Score of different age (>60 and <60) at all time points after surgery. (B) Changes of Forgotten Joint Score of different BMI (>30 and <30) at all time points after surgery. (C) Changes of Forgotten Joint Score of different duration of onset (>3 ys and <3 ys) at all time points after surgery.

The postoperative FJS score at each time point (1 month, 6 months, 12 months, 24 months, and 36 months) was used as the dependent variable, and the significant variables in Table 2 (age, BMI, and duration of onset before surgery) were used as independent variables for the multiple linear regression analysis. The results revealed that age, BMI, and duration of onset before surgery were all predictors of FJS after surgery (Table 3). Interestingly, in our study older patients (age > 60) were found to be a positive predictor of good outcome for the FJS; Fig. 2A highlighted that age has no significantly influence on FJS at 1 and 6 months, while it was positively associated with FJS from 12 months after surgery (Table 3 and Fig. 2A). Lower BMI (BMI < 30) was found to be a positive predictor of good outcome for the FJS after surgery; Fig. 2B highlighted that the relationship between FJS and BMI became significant from 12 months where increased FJS is clearly associated with decreased BMI (BMI < 30) (Table 4 Table 3 and Fig. 2B, *P* < 0.05). Furthermore, there was a negative correlation between duration of onset before surgery and FJS score from 12 months after surgery (Table 3 and Fig. 2C).

**Table 3:** Multiple linear regression results of FJS and other factors at various time points after medial unicompartmental knee arthroplasty

	1 month				6 month			
	β	SE(β)	*t*	*P*	β	SE(β)	*t*	*P*
Constant	29.368	10.951	2.682	0.011	65.228	9.248	7.053	0.000
Age	6.525	4.560	1.431	0.162	−4.306	3.318	−1.298	0.203
BMI	0.284	4.441	0.064	0.949	−0.474	3.344	−0.141	0.888
Duration	3.308	4.560	0.725	0.473	3.847	3.335	1.153	0.257

	12 month				24 month				36 month			
	β	SE(β)	*t*	*P*	β	SE(β)	*t*	*P*	β	SE(β)	*t*	*p*
Constant	57.512	6.936	8.288	0.000	66.021	9.745	6.775	0.000	66.730	9.266	7.201	0.000
Age	12.627	3.417	3.695	0.001	14.526	3.186	4.559	0.000	15.468	3.450	4.484	0.000
BMI	−4.892	2.603	−1.879	0.009	−9.727	3.276	−2.969	0.005	−9.162	3.263	−2.807	0.008
Duration	4.869	3.253	1.497	0.004	2.589	3.341	0.775	0.004	1.140	3.054	0.373	0.007

BMI, body mass index; FJS, forgotten joint score; Duration, duration of onset before surgery

β, regression coefficient; SE(β), standardized regression coefficient.

## Discussion

The outcomes utilizing FJS in the literature to evaluate patients' subjective feelings after joint replacement are rare, especially regarding UKA. To the best of our knowledge, there are few studies that analyze the improvements of FJS for UKA over a 3‐year follow‐up interval^9^, ^10^. Based on our results, we found that there was improvement of FJS till 12 months post‐operation. After that there were no more improvements. The patients achieved platform stage in joint awareness from 12 months to 36 months after UKA surgery.

FJS is a new concept to evaluate the outcomes of joint replacement, representing the patient's ability to forget artificial joints in daily life, which, in some ways, is the ultimate goal to evaluate the postoperative satisfaction of patients^6^. There were many patient reported outcome measures (PROs) that evaluate patients' postoperative status today, but studies by different authors often yield inconsistent outcomes. A previous study compared the Oxford Knee Score (OKS) and the EQ‐5D of 23,393 TKA patients and 505 UKA patients and found no difference between them^11^. However, the Norwegian Arthroplasty Register compared 972 TKA cases with 372 UKA cases using the Knee Injury and Osteoarthritis Outcome Score (KOOS), the EW‐5D and the visual analogue scale (VAS), and found that UKA group patients had better PROs results^12^. As a result, these PROs scores are unable to assess top‐end differences after joint replacement, reaching a ceiling effect, which is not a restriction to FJS scores. In addition, Behrend *et al*. found that even in healthy people the average score of FJS was 82.5 rather than 100, indicating that it could accurately differentiate highly functional groups with joint arthroplasty^6^. It was for these reasons that we chose the FJS scores to evaluate the patients reported outcomes following UKA.

Kim *et al*. compared the FJS scores between 100 TKAs and 100 UKAs at an average follow‐up of 2 years, and found that the FJS of patients who had undergone UKA (67.3 ± 19.8) was significantly higher than patients who had undergone TKA (60.6 ± 16.6)^10^. Similarly, in a recent study, Zuiderbaan *et al*. compared the postoperative FJS of 65 UKAs with 65 TKAs, and found that the FJS of UKA (73.9 ± 22.8) was significantly higher than that of TKA (59.3 ± 29.5) at 1 year after surgery, while the outcomes at 2 years after surgery were slightly improved compared with that at 1 year after surgery. The authors concluded that UKA is more likely than TKA to forget the presence of artificial joints^9^. Their values are very close to the values of our study obtained at 12 and 24 months after surgery. Our study also supported an eventual plateau of UKA in joint awareness for 12–36 months after surgery, which is similar to the study of Zuiderbaan *et al*.^9^. Our current study also demonstrated dramatic improvement in FJS in the short term (6 months) after medial UKA, and kept improving until 12 months after surgery. Even at the peak of 36 months after surgery, postoperative FJS scores of UKA were still slightly lower than those of healthy people (79.3–86.6), which suggested that even partial joint replacements like UKA, which retain ligaments and bone mass, cannot fully restore normal knee function^6^.

Another important finding of the present study is the predictive value of preoperative BMI for outcome assessment based on the FJS‐12. We found that BMI was negatively correlated with FJS, suggesting that patients with greater weight had more difficulty in forgetting their UKA. Most researchers have shown that obesity will lead to higher infection rates and inferior prosthetic survival^6^, ^13^, ^14^. However, there is no consensus on the relationship between obesity and postoperative knee function after UKA. Murray *et al*. assessed the relationship between BMI and postoperative function in 2,438 patients with medial Oxford mobile‐bearing UKA, and found no significant relationship between BMI and postoperative knee function^15^. However, Thompson *et al*. evaluated 229 patients with UKA and found that BMI >35 was correlated with lower KSS scores than patients with BMI <35^16^. According to our results, when deciding to perform UKA in obese patients, the effects of obesity on FJS need to be taken into consideration.

Interestingly, in this study we found older patients (aged >60 years) and with longer duration of onset before surgery (>3 years) to be a positive predictor of good outcome for the FJS‐12. One possible explanation for this might be that as activity levels naturally decline with age, the awareness of the joint during activities may also decrease. Dunbar *et al*.^17^ believed that patients with chronic diseases are more likely to be satisfied with the improvement of pain and function after TKA treatment. However, patients with more recent disease and shorter duration of symptoms prior to TKA tend to be less satisfied. In contrast, patients with chronic and systemic diseases seem to be more receptive to a decline in health. In addition, in older people, general health problems tend to overshadow minor joint‐related damage, which may be the reason why older patients with longer duration of onset are more likely to forget about artificial joints after UKA surgery.

Another finding of our study was that gender and Kellgren‐Lawrence grade (III or IV) of the medial compartment before surgery had no significant influence on the FJS of patients following medial UKA. This finding may be related to the small sample size in our study. Finally, the findings of our study offer straightforward clinical application that surgeons can use to educate their patients before undergoing UKA.

There are some weaknesses in this research. First of all, this study is cross‐sectional research without utilizing a single patient cohort to confirm our research. Prospective cohort study is necessary in the future. Although potential confounding variables were considered in this study, it is possible that other factors influenced the observed outcomes; for example, older patients may have impaired memory. Secondly, FJS scores have the advantage of not being influenced by ceiling effect. However, this method can only be applied postoperatively rather than preoperatively, because it is used to assess the ability of patients to forget artificial joints in their daily life after surgery. Thirdly, the follow‐up time is short, and the results of long‐term follow‐up are still unknown. Maybe with the extension of follow‐up time, the FJS score will decrease due to the progression of OA in other compartments or the wear of prosthesis.

### *Conclusion*

This study demonstrates the temporal relationship of medial UKA and FJS. In addition, preoperative BMI, age, and duration of onset before surgery were predictive for FJS outcome evaluation, while the gender and Kellgren‐Lawrence grade (III or IV) of the medial compartment before surgery had no influence on FJS of patients who have undergone UKA. This information can be used for improved patient selection and our study may provide an accurate post‐operative expectation for patients undergoing UKA.
